# Loss of Vision Dominance at the Preresponse Level in Tinnitus Patients: Preliminary Behavioral Evidence

**DOI:** 10.3389/fnins.2019.00482

**Published:** 2019-05-14

**Authors:** Zhicheng Li, Ruolei Gu, Min Qi, Jintian Cen, Shuqi Zhang, Jing Gu, Xiangli Zeng, Qi Chen

**Affiliations:** ^1^Department of Otolaryngology-Head and Neck Surgery, Third Affiliated Hospital of Sun Yat-sen University, Guangzhou, China; ^2^Department of Psychology and Center for Studies of Psychological Application, South China Normal University, Guangzhou, China; ^3^Key Laboratory of Behavioral Science, Institute of Psychology, Chinese Academy of Sciences, Beijing, China

**Keywords:** tinnitus, cross-modal interference, vision dominance, attention, behavioral research

## Abstract

At present, the mechanisms underlying changes in visual processing in individuals with tinnitus remain unclear. Therefore, we investigated whether the vision dominance of individuals with tinnitus disappears at the preresponse level through behavioral study. A total of 38 individuals with tinnitus and 31 healthy controls completed a task in which they were asked to attend to either visual or auditory stimuli while ignoring simultaneous stimulus inputs from the other modality. We manipulated three levels of congruency between the simultaneous visual and auditory inputs: congruent (C), incongruent at the preresponse level (PRIC), and incongruent at the response level (RIC). Thus, we differentiated the cross-modal conflict explicitly into the preresponse (PRIC > C) and response (RIC > PRIC) levels. The results revealed no significant difference in the size of the preresponse level conflict between the auditory attention and visual attention conditions in tinnitus group. In brief, the preresponse level of individuals with tinnitus showed a loss in vision dominance. This may be due to the reduced interference of visual information in auditory processing.

## Introduction

Vision and audition are important functions through which individuals obtain information about the external environment. When individuals attend to information from one sensory modality, they tend to “look without seeing” or “listen without hearing” to the information received by other sensory modalities. However, the information received by the non-attended modality affects the information processing of that received by the attended modality, and this phenomenon is known as cross-modal interference. Previous studies have reported that when the input information of visual and auditory modalities is inconsistent, the processing time is prolonged and the accuracy of responses to attended stimuli decrease (Colavita, 1974; Chen and Zhou, 2013). Moreover, visual distractors cause more interference to auditory processing than auditory distractors do to visual processing (as vision dominance) at the preresponse level, but auditory distractors cause more interference to visual processing than visual distractors do to auditory processing (as audition dominance) at the response level (Repp and Penel, 2004; Kato and Konishi, 2006; Mayer et al., 2009; Koppen et al., 2009; Chen and Zhou, 2013).

Tinnitus, a subjective auditory experience, emerges independent of external stimuli, and its occurrence and maintenance require attentional resources (Roberts et al., 2013). Previous studies have shown that visual processing in individuals with tinnitus is significantly worse than that of healthy controls (Stevens et al., 2007; Heeren et al., 2014; Araneda et al., 2015a, b; Li et al., 2018), and is more susceptible to cross-modal interference of auditory information (Araneda et al., 2015b). Meanwhile, the decrease in signal detection (the early stage of cognitive processing) might underlie the impaired visual processing in individuals with tinnitus (Li et al., 2018). With this in mind, the “visual dominance effect” (i.e., allocating more attentional resources to visual rather than auditory information, which leads to stronger interference of visual distractors on auditory targets) at the preresponse level might function abnormally in individuals with tinnitus. Clarifying this problem will facilitate further understanding of the effect of tinnitus on individuals’ cognitive processing (particularly cross-channel information integration), and will provide a reference for improving strategies concerning the evaluation and management of tinnitus. However, the changes and mechanisms of visual processing in individuals with tinnitus are unclear.

**FIGURE 1:**
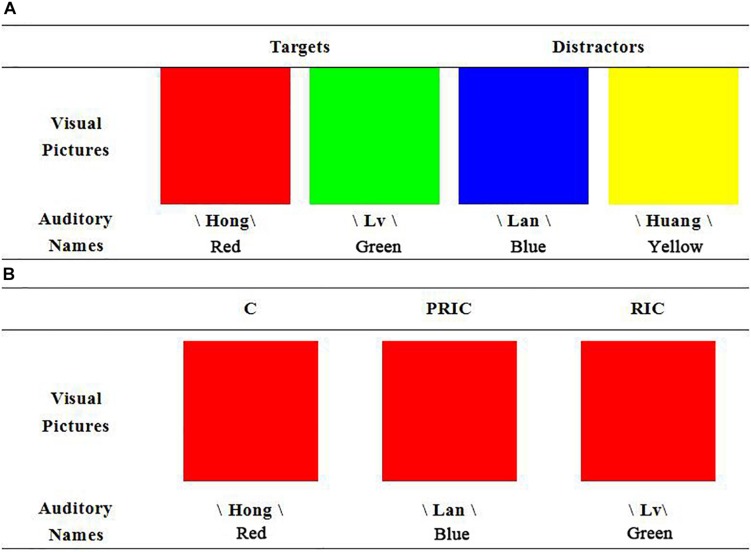
Exemplar stimuli **(A)** and design **(B)** in experiment. **(A)** Two color pictures and their verbal sounds served as targets. Another two color pictures and their verbal sounds served as distractors. **(B)** Examples of the manipulation of the three levels of congruency are provided for the situation in which the visual modality was attended. In the C condition, the auditory name and visual picture refer to the same target. In the PRIC condition, the auditory name refers to the distractors and the visual picture refers to the target. In the RIC condition, the auditory name and visual picture refer to different targets. The auditory names were played in Mandarin, like /Hong/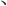/Lv/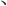/Lan/, and /Huang/.

The current study aimed to investigate whether the vision dominance of individuals with tinnitus disappears at the preresponse level. To this aim, we used the experimental design of Chen and Zhou (2013), which could help investigate cross-modal conflict at the preresponse and response levels. Participants simultaneously received visual and auditory stimuli (i.e., cross-modal inputs), and were asked to attend to one modality and ignore the other. All experimental stimuli were presented as one of four colors (red/green/blue/yellow), two of which (red and green) were targets, while the other two (blue and yellow) were distractors (Figure 1A); only stimuli in the “target” category required press the button. The task included the two following factors: modality (attending to visual/auditory stimuli) and congruency (cross-modal congruency, C; incongruent at preresponse level, PRIC; incongruent at both the preresponse and response levels, RIC). In the C condition, bimodal inputs referred to the same target (e.g., participants saw a red block and heard “red”). In the PRIC condition, the stimulus in the attended channel (visual/auditory) was a target, but the stimulus in the unattended channel was a distractor that did not require a response (e.g., participants saw a red block but heard “blue”); therefore, the cross-modal conflict emerged at the preresponse level but not the response level. In the RIC condition, the stimuli in the attended channel and the unattended channel were two different targets that required different responses (e.g., participants saw a red block but heard “green”) (Figure 1B); therefore, the cross-modal conflict emerged at both the preresponse and response levels. According to Chen and Zhou (2013), the classical findings in this task are that visual distractors cause larger preresponse-level interference (PRIC > C in reaction time) to auditory processing than vice versa, while auditory distractors cause larger response-level interference (RIC > PRIC in reaction time) to visual processing than vice versa.

Based on these previous studies, we hypothesized that the vision dominance of individuals with tinnitus would disappears at the preresponse level, and there would be no significant difference between the interference of visual information in auditory processing and vice versa. In line with this hypothesis, we found that there was no significant difference in preresponse-level interference between the modalities attended. This study did not have any specific predictions concerning the disappearance of visual dominance.

## Methods

The experimental protocol was reviewed and approved by the Ethics Committee of the Third Affiliated Hospital of Sun Yat-sen University [approval number: (2018) 02-358-01]. All enrolled participants were required to sign an informed consent form.

### Participants

Individuals with tinnitus admitted to the Outpatient Department of Otorhinolaryngology, the Third Affiliated Hospital of Sun Yat-sen University due to tinnitus as a major complaint were enrolled (21 men, 17 women, mean age = 28.23 ± 6.20 years). All individuals were diagnosed as having chronic subjective tinnitus, without hyperacusis, and the pure-tone threshold from 125 to 8000 Hz, including the semioctave range, were ≤40 dB HL. Healthy controls, who had no history of hearing loss (both pure-tone threshold ≤25 dB HL), tinnitus, dizziness, or other ear diseases, were recruited from online and poster adverts at the Sun Yat-sen University (16 men, 15 women, mean age = 22.63 ± 2.24 years). All participants were right-handed, had normal or corrected-to-normal vision (5.0 or above in the Logarithmic Visual Acuity Chart), and no color blindness or weakness. No participant had a history of neurological or psychiatric disorders.

### Experimental Design and Stimuli

We adopted a 2 (group: tinnitus and control groups) × 2 (modality attended: Attend-Visual and Attend-Auditory) × 3 (congruency: C, PRIC, and RIC) hybrid design.

Visual stimuli (color blocks) were presented on an LED monitor at a viewing distance of 70 cm. Auditory stimuli (verbal pronunciations) were voice recordings of a male speaker and were delivered binaurally via stereo headphones. The volume was adjusted for each participant such that the auditory stimuli could be clearly heard.

The experimental task was to judge whether the attended color block or verbal pronunciation was red or green. Throughout the experiment, all color blocks and verbal pronunciations were potential targets that required responses. Participants used the index and middle fingers of their right hand to respond by pressing one key on the response box for red or another key for green. The mapping between the two response keys and red versus green color was counterbalanced across participants.

### Procedure

The stimuli were presented using a hybrid design in which the attended modality was blocked, and the C, PRIC, and RIC trials were mixed randomly within each block. In each block, participants were asked to focus on either the visual or auditory stimuli while ignoring stimuli from the other modality. They were instructed to fixate on the central cross throughout the experiment without moving their eyes. In each trial, a color block and verbal pronunciation were simultaneously presented for 300 ms. Each of the six experimental conditions had 48 trials, resulting in a total of 384 trials (288 experimental trials and 96 null trials). In a null trial, only the central fixation cross was displayed. For the visual attention condition and auditory attention condition, respectively, null trials and C, PRIC, and RIC trials were randomly mixed and then divided into 24 test blocks. Each block comprised 8 trials and lasted for 20 s. The visual attention condition and auditory attention condition blocks were alternated. Each block started with a 2-s visual instruction about which modality was to be attended.

### Statistical Analyses

Within each of the six experimental conditions, omissions, incorrect responses, and trials with reaction times (RTs) three standard deviations away from the mean RT were excluded from further analyses. The mean RTs of the remaining trials were subsequently calculated. Normally distributed data were analyzed using repeated measures analysis of variance (ANOVA) and *t*-tests. Otherwise, median and quartile ranges were presented, and differences were tested using Wilcoxon Signed Ranks Test. *p* < 0.05 was considered statistically significant.

## Results

**FIGURE 2:**
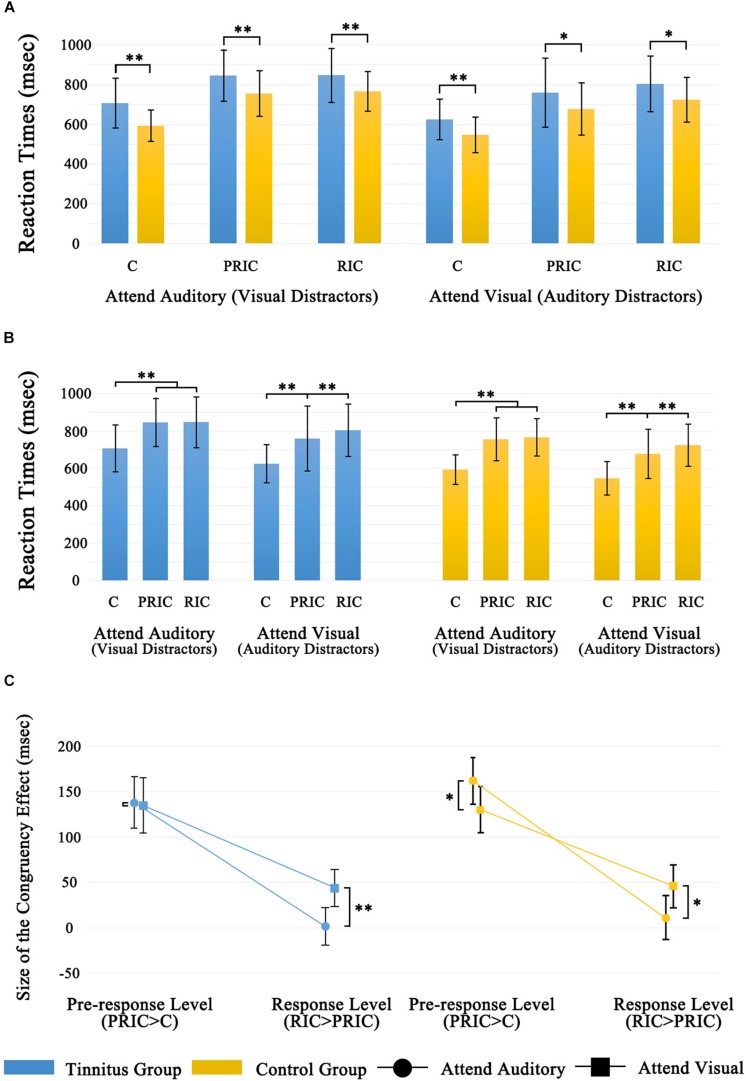
Behavioral results of Experiment. **(A)** Inter-group differences of the six experimental conditions. **(B)** Intra-group differences of the six experimental conditions. **(C)** Inter-group differences of sizes of cross-modal conflict at the preresponse (PRIC > C) and response (RIC > PRIC) levels are shown as a function of the attended modality. *p < 0.05; ^∗∗^*p* < 0.01.

The 2 (group: tinnitus and control groups) × 2 (modality attended: Attend-Visual and Attend-Auditory) × 3 (congruency: C, PRIC, and RIC) repeated measures ANOVA showed a significant main effect of Group [*F*(1,67) = 9.15, *p* = 0.004], whereby the tinnitus group had significantly longer RTs than those of the control group, irrespective of the attended modality or congruency condition (*p* < 0.05; Figure 2A).

Moreover, there was a significant main effect of attended modality [*F*(1,67) = 76.94, *p* < 0.001], whereby RTs to the visual targets were significantly shorter than those to the auditory targets. There was also a main effect of congruency [*F*(2,134) = 284.53, *p* < 0.001]; further pairwise comparisons using Bonferroni correction indicated that RTs in the PRIC condition were significantly longer than those in the C condition (*p* < 0.001), and RTs in the RIC condition were significantly longer than those in the PRIC condition (*p* = 0.02). This result indicated significant cross-modal conflicts at both the preresponse and response levels.

There was a significant interaction between the attended modality and congruency [*F*(2,134) = 7.44, *p* = 0.001], and planned *t*-tests on simple effects indicated that the preresponse level conflict was significant both in the auditory attention condition (*p* < 0.001) and visual attention condition (*p* < 0.001). Conversely, the response level conflict was only significant in the visual attention condition (*p* = 0.001), and not in the auditory attention condition (*p* = 0.40). There were no significant interactions between attended modality and group [*F*(1,67) = 1.38, *p* = 0.25], consistency and group [*F*(2,134) = 0.50, *p* = 0.61], attended modality, consistency, and group [*F*(2,134) = 1.80, *p* = 0.17]. This indicated that the pattern between attended modality and consistency were similar between the tinnitus group and control group (Figure 2B).

Within the control group, the size of the preresponse level conflict was significantly larger when the auditory modality was attended vs. when the visual modality was attended (*p* = 0.02), whereas the size of the response level conflict was significantly larger when visual modality was attended vs. when the auditory modality was attended (*p* = 0.04) (Figure 2C, right). Within the tinnitus group, the size of the preresponse level conflict was not significantly different between the auditory attention and visual attention conditions (*p* = 0.85) (Figure 2C, left).

For both the auditory attention and visual attention conditions, further independent samples *t*-tests did not reveal any significant between-group differences in the size of the congruency effect at the preresponse and response levels groups (*p* = 0.24, 0.59, 0.82, and 0.91, respectively).

## Discussion

The brain can process a limited amount of information per unit time; therefore, the brain needs to allocate limited attention resources to process the most pertinent information in complex situations (Mishra and Gazzaley, 2012). The occurrence and maintenance of tinnitus both require attentional resources, which eventually affects cognitive processing (Roberts et al., 2013). The present study found that the RTs of the tinnitus group in both experimental conditions were significantly longer than those of the control group, thus supporting the reduction of visual and auditory processing in individuals with tinnitus.

More importantly, we found that the vision dominance of individuals with tinnitus disappeared at the preresponse level, as hypothesized. Although the size of the congruency effect at the preresponse level did not show significant differences between the tinnitus and control groups, it reduced from 162 ms in the control group to 138 ms in the tinnitus group in the auditory attention condition, while it maintained a similar effect in the tinnitus (135 ms) and control (130 ms) groups in the visual attention condition. Therefore, we speculate that the disappearance of visual dominance at the preresponse level in individuals with tinnitus is mainly due to the reduced interference of visual information in auditory processing. This finding also indicates that the auditory modality may demand a greater allocation of attention, which consequently weakens the interference of the visual modality.

However, we must also realize that tinnitus may be a product of brain dysfunction (Elgoyhen et al., 2015), and brain dysfunction may also affect other cognitive processes, other than auditory processing. Thus, whether the reduction of visual processing in individuals with tinnitus is due to tinnitus, or, like tinnitus, still needs to be further explored. Moreover, the default mode network is involved in visual dominance at the preresponse level (Chen and Zhou, 2013), and many brain regions and neural connections in the default mode network of individuals with tinnitus have been found to exhibit changes (Elgoyhen et al., 2015). We aim to explore the relationship between the disappearance of visual dominance at the preresponse level and the change in the default mode network in our follow-up EEG and imaging studies. Furthermore, this study only focused on individuals with chronic subjective tinnitus. Therefore, the results of the study cannot be extended to other groups of individuals with tinnitus. Follow-up studies should gradually incorporate different groups of individuals of tinnitus, such as those with acute tinnitus or objective tinnitus, to further clarify this viewpoint.

## Conclusion

The preresponse level of individuals with tinnitus revealed a loss of vision dominance, which may be due to the reduced interference of visual information in auditory processing. Further studies are warranted to verify our findings and to explore the neural mechanisms underlying behavioral changes using EEG and imaging techniques.

## Author Contributions

ZL designed and performed the experiments, analyzed the data, and wrote the manuscript. RG analyzed the data and perfected the manuscript. XZ modified the research approach and chose the patients. QC directed and modified the research approach and provided the critical revision. ZL, RG, XZ, and QC discussed the results and implications and commented on the manuscript at all stages. MQ, JC, SZ, and JG in charge of preliminary screening and contacted with subject.

## Conflict of Interest Statement

The authors declare that the research was conducted in the absence of any commercial or financial relationships that could be construed as a potential conflict of interest.
